# Quantitative Ultrasound Texture Analysis of Breast Tumor Responses to Chemotherapy: Comparison of a Cart-Based and a Wireless Ultrasound Scanner

**DOI:** 10.3390/jimaging12030129

**Published:** 2026-03-13

**Authors:** David Alberico, Maria Lourdes Anzola Pena, Laurentius O. Osapoetra, Lakshmanan Sannachi, Joyce Yip, Sonal Gandhi, Frances Wright, Michael Oelze, Gregory J. Czarnota

**Affiliations:** 1 Physical Sciences, Sunnybrook Research Institute, Toronto, ON M4N 0A4, Canada; david.alberico@sunnybrook.ca (D.A.); marialourdes.anzolapena@sunnybrook.ca (M.L.A.P.); laurentiusoscar.osapoetra@sunnybrook.ca (L.O.O.); lakshmanan.sannachi@sunnybrook.ca (L.S.); waiszejoyce.yip@sunnybrook.ca (J.Y.); 2 Division of Medical Oncology, Department of Medicine, Sunnybrook Health Sciences Centre, Toronto, ON M4N 0A4, Canada; sonal.gandhi@sunnybrook.ca; 3 Department of Medicine, University of Toronto, Toronto, ON M5S 1A1, Canada; 4 Department of Division of Surgical Oncology, Department of Surgery, Sunnybrook Health Sciences Centre, Toronto, ON M4N 0A4, Canada; frances.wight@sunnybrook.ca; 5 Department of Surgery, University of Toronto, Toronto, ON M5S 1A1, Canada; 6 Department of Electrical and Computer Engineering, University of Illinois Urbana-Champaign, Champaign, IL 61801, USA; oelze@illinois.edu; 7 Department of Radiation Oncology, Sunnybrook Health Sciences Centre, Toronto, ON M4N 0A4, Canada; 8 Department of Medical Biophysics, University of Toronto, Toronto, ON M5S 1A1, Canada; 9 Department of Radiation Oncology, University of Toronto, Toronto, ON M5S 1A1, Canada

**Keywords:** breast cancer, quantitative ultrasound, texture analysis, wireless ultrasound

## Abstract

This study assessed the level of agreement between quantitative ultrasound (QUS) feature estimates derived from ultrasound images of breast tumors in women with locally advanced breast cancer (LABC) produced using a cart-based and a handheld ultrasound system. Thirty LABC patients receiving neoadjuvant chemotherapy were imaged at two separate times: a pre-treatment ‘baseline’ time point, and four weeks after the start of chemotherapy. Three sets of QUS features were produced using the reference phantom technique, one for each imaging time and a third set calculated by taking the differences in feature estimates between times. Cross-system statistical testing using the Wilcoxon signed-rank test was performed for each feature set to assess the level of feature estimate agreement between ultrasound systems. The Bland–Altman method was employed to graphically assess feature sets for systematic skew. The range of *p*-values was 4.50 × 10^−11^ to 0.277 for the baseline features, 2.77 × 10^−5^ to 0.865 for the week 4 features, and 2.03 × 10^−9^ to 1 for the feature differences. For the feature differences, all five of the primary QUS features (MBF, SS, SI, ASD, AAC) were found to be in agreement between the two scanner types at the 5% confidence level. For the baseline feature set and week 4 feature set, 0 out of 5 and 3 out of 5 of the primary features were found to be in agreement, respectively. Of the 20 QUS texture features examined, the number and proportion of the total for each feature set which were found to have statistically significant similarity in their sample medians at the 5% confidence level were as follows: 2 out of 20 (10%) for the baseline features; 17 out of 20 (85%) for the week 4 features; and 12 out of 20 (60%) for the feature differences. The specific texture features found to be in agreement varied between QUS-specific feature sets. Overall, a moderate level of agreement between sets of feature differences produced using the two systems was demonstrated.

## 1. Introduction

Quantitative ultrasound (QUS) encompasses techniques for obtaining quantitative estimates of tissue properties using ultrasound imaging. This is achieved by analyzing ultrasound radiofrequency (RF) data using signal processing techniques to calculate estimates of the normalized power spectrum (NPS) and backscatter coefficient spectrum (BSC) for the tissue of interest and then extracting spectral features which contain information about the tissue’s microstructural properties. The application of these techniques has been investigated in a variety of clinical contexts, such as distinguishing between benign and malignant breast tumors [1].

Another clinical application of QUS techniques is for monitoring the effects of chemotherapy in patients with locally advanced breast cancer (LABC) [2]. This is done by imaging the tumor and collecting ultrasound RF data at multiple times during the course of patient chemotherapy regimens, extracting QUS features and texture derivative features at each time, and then calculating the change in feature estimates between time points and a pre-treatment baseline. Prediction models can then be constructed using these feature differences as training data to identify which changes in the QUS features correspond to changes in tissue microstructure associated with cell death and, consequently, significant tumor response to chemotherapy. Clinical trials have demonstrated that it is possible to use these techniques to train QUS-based models capable of predicting tumor response in LABC patients with accuracies as high as 92% [3].

Previous studies of QUS-based response prediction for LABC have focused on traditional cart-based clinical ultrasound systems. It is desirable that these methods be reproducible using a variety of different ultrasound systems and transducers, and consequently the reproducibility of QUS feature estimates of tissue has been the subject of several previous investigations [2,4,5,6]. A 2010 study by Wirtzfeld et al. performed ultrasound imaging on tumors grown in rat models and made measurements of the tissue backscatter coefficient. They imaged six animals using three clinical and one preclinical ultrasound system using a total of nine different transducers and reported broad agreement in measurements between systems and transducers. A follow-up study published in 2015 compared ultrasound backscatter coefficient estimates of tumors grown from two breast cancer cell lines with three different ultrasound devices again reported good agreement across imaging systems. In 2020 Sannachi et al. [7] performed imaging on a cohort of 24 women presenting with breast cancer with two different clinical ultrasound scanners in order to investigate the level of agreement in estimates of QUS features and texture features and determine potential impact on tumor response prediction models. That study did not reveal any significant impact on feature estimates or model performance.

Previous inter-system and inter-transducer comparisons of QUS feature estimates have focused on comparing scanners with similar characteristics and imaging performance. Handheld ultrasound systems would bring certain advantages to potential clinical applications of QUS techniques, but their relatively lower scan line densities and sampling rates compared to traditional cart-based clinical scanners could potentially impact the reproducibility of QUS techniques. The objective of the present study was to quantify the impact of system differences on QUS feature estimates for a cohort of LABC patients imaged at two different times, a pre-treatment baseline time and at four weeks after the initial administration of chemotherapy, produced using two different ultrasound systems, a traditional cart-based system and a portable handheld scanner, whose imaging characteristics are representative of the handheld-cart-based performance gap.

The devices under study were the Sonix RP (RP) (Ultrasonix Medical Corporation, Richmond, BC, Canada) and the Clarius L15HD (CL15) portable ultrasound scanner (Clarius Mobile Health, Vancouver, BC, Canada). The Clarius L15HD is a portable linear-array scanner which has RF data export functionality. The Sonix RP was selected for comparison because QUS feature estimates obtained from ultrasound RF data captured using this system have been used for QUS response monitoring of LABC in previous studies [2,3,7,8].

A cohort of 30 women with clinically proven LABC participated. Ultrasound scans were performed at two time points: a pre-treatment scan (‘baseline’) and at four weeks after the start of chemotherapy (‘week 4’). Imaging was performed with both the Sonix RP and Clarius L15HD ultrasound systems. After calculating QUS feature estimates at the baseline and week 4 time points, the differences in feature estimates were calculated and compared in order to assess the level of cross-system agreement using statistical and graphical analysis.

## 2. Materials and Methods

### 2.1. Ultrasound Systems and Scanning Protocol

Imaging was performed using both a handheld ultrasound system and a cart-based ultrasound system. The handheld system was the Clarius L15HD portable ultrasound scanner system, which uses an in-built linear-array transducer. The cart-based system selected for comparison was the Sonix RP using an L14-5W/60 linear array transducer (Ultrasonix Medical Corporation, Richmond, BC, Canada). System and transducer properties are summarized in Table 1.

A total of 30 patients recruited from the Louise Temerty Breast Cancer Centre (Sunnybrook Health Sciences Center, Toronto, ON, Canada) consented to participate in the study. All patients had histologically or cytologically confirmed breast carcinoma of stage I-IV which had not been previously treated with any first-line therapy, and all were subsequently treated with neoadjuvant chemotherapy according to standard institutional practice. Only patients with a primary mass measuring at least 1 cm in diameter were considered for participation. Women with breast implants were excluded from consideration for the study. Patients selected for analyses were classified as either responders or non-responders to chemotherapy as per RECIST 1.1 criteria [9] based on tumor size changes and histopathological characteristics obtained from post-treatment pathology reports.

For each patient, breast imaging was performed twice: once at the pre-treatment ‘baseline’ time point and again four to five weeks after the first administration of chemotherapy. Scans were performed by one of the four ultrasound operators associated with the study. All of the operators were instructed to use the same scanning procedure in order to reduce inter-operator bias. Collection of ultrasound image data was performed as follows. Using each ultrasound system, reference images were taken along the tumor’s longest dimensions in a pair of perpendicular imaging planes. A panning scan followed, with the transducer oriented radially across the tumor volume with respect to the nipple. Transducer focus position, transmit–receive frequency, time gain compensation, and other imaging parameters remained consistent across operators, scans, and patients. During each imaging session, both ultrasound systems under consideration were used to image the tumor one after the other within a fifteen-minute timeframe.

### 2.2. Ultrasound Data Analysis

For each set of ultrasound image data, an ultrasound reader hand-selected a complex polygonal region of interest (ROI) around the tumor boundary using available clinical ultrasound B-mode images as a referential aid. The ultrasound reader was not blinded with respect to patient identity, system type, or scan time point. Frames where the tumor margins were clearly visible were manually selected, with an attempt being made to select frames which were spread out across the tumor volume. For each set of image data, between 4 and 6 frames of ultrasound RF data were segmented and subsequently analyzed. ROI selections were subsequently confirmed by a participating radiologist with 10+ years of ultrasound research experience. ROI segmentation was done using purpose-built software written in MATLAB (The MathWorks Inc. MATLAB Version: 9.1.0.1012177 (R2016b). Available at https://www.mathworks.com, accessed on 1 January 2025. Custom code available from the authors upon request).

QUS feature determination was performed for all clinical images following procedures described in previous publications [6,7]. ROIs were subdivided into square data blocks 2 mm in dimension, corresponding to 10 times the ultrasound wavelength at the transducer’s center frequency. Data blocks were selected in an overlapped tiling pattern such that 80% of the area of each block overlapped with the previous block. The average power spectral density for each block was calculated by applying a Hanning window with a length of 104 samples and a full width at half maximum of 51 samples before taking the magnitude of the fast Fourier transform (FFT) of the signal and then averaging over all RF lines within the block. The resulting backscattered power spectrum was normalized using the reference phantom technique [10] with ultrasound image data collected from a homogenous reference phantom.

The tissue-mimicking phantom used for reference normalization was made with scatterers of diameters ranging from 5 to 30 μm suspended in a gelatinous substrate, prepared by the Medical Physics Department at the University of Wisconsin, Madison, WI, USA. The Department of Electrical and Computer Engineering, University of Illinois, Urbana, IL, USA, made measurements of the phantom’s acoustic properties. Measurements of attenuation coefficients and the medium speed of sound were done using the insertion loss and arrival time difference methods [11,12]. The attenuation coefficient was 0.7861 dB×cm^−1^×MHz^−1^ and the speed of sound was 1488 m/s. The phantom’s backscatter coefficient spectrum was measured using a plane-reflector-based method [13,14].

In order to compensate for attenuation, attenuation within the tumor volume (local attenuation) and attenuation within the intervening tissue layers between the tumor boundary and the skin surface were calculated separately before combining them to estimate the total attenuation. A breast tissue attenuation coefficient of 1 dB×cm^−1^×MHz^−1^ was assumed for the purpose of estimating and correcting for total acoustic attenuation, based on available literature on the acoustic properties of tissue [15]. For estimating local attenuation within the tumor volume itself, a reference-phantom-based spectral-difference method was used [16]. The log-transformed reference-normalized power spectrum was calculated for each windowed data block within the ROI. The power at each frequency in the transducer frequency band was then averaged across all windows at the same axial depth to obtain the average log-transformed power at each frequency at each depth within the tumor. An estimate of the local attenuation coefficient was obtained by performing a linear regression of power versus depth for each frequency, dividing the resulting slope by the corresponding frequency, and then averaging across all frequencies. The total attenuation coefficient was then used to perform point compensation of the normalized power spectrum for each windowed data block.

The QUS features under consideration were the mid-band fit (MBF), spectral slope (SS), spectral intercept (SI), average scatterer diameter (ASD), and average acoustic concentration (AAC). The MBF, SS, and SI were estimated by performing linear regression analysis of the attenuation-compensated log-compressed normalized power spectrum over the overlapping region of the −6 dB frequency bandwidths of the two ultrasound systems (5.1–8 MHz). ASD and AAC were obtained from estimates of the BSC. The sample BSC spectra were calculated from the normalized power spectrum.

$$\sigma_{s}\left(f,z\right)=\frac{W_{s}\left(f,z\right)}{W_{r}\left(f,z\right)}\sigma_{r}(f)e^{4\left(\alpha_{m}\left(f\right)z_{m}+\alpha_{i}\left(f\right)z_{i}-\alpha_{r}\left(f\right)z\right)}$$

Here, z is the axial depth of the current data block relative to the transducer ($z=z_{i}+z_{m})$; $z_{i}$ is the axial distance between the transducer and the edge of the ROI contour defining the tissue-tumor interface (the distance relevant for total attenuation correction); $z_{m}$ is the axial depth of the data block with respect to the ROI (the distance relevant for local attenuation correction within the tumor volume itself); f is the frequency in MHz; $W_{s}\left(f,z\right)$ and $W_{r}\left(f,z\right)$ are respectively the backscattered power spectra of the sample and the reference; $\sigma_{s}\left(f,z\right)$ and $\sigma_{r}(f)$ are the backscatter coefficients of the sample and the reference; $\alpha_{m}(f)$, $\alpha_{m}(f)$, and $\alpha_{r}(f)$ are the attenuation coefficients for the tumor, background tissue, and tissue-mimicking phantom used as a reference. The estimated sample BSC was compared to the BSC calculated based on scattering theory, BSC ($\sigma_{theory}$), using a least-squares method. An estimate of the average scatterer diameter was obtained by conducting a parameter sweep to find the scatterer size which minimized the difference between the measured and theoretical BSC. The theoretical BSC assumed scatterers with a Gaussian form factor [17,18], given by:

$$\sigma_{theory}\left(f\right)=\frac{\pi^{4}}{36c^{4}}f^{4}a_{eff}^{6}\bar{n}\gamma_{0}^{2}{FF}_{G}(f,a_{eff})$$

$${FF}_{G}\left(f,a_{eff}\right)=e^{-0.827(\frac{2\pi f}{c}a_{eff})}$$

where ${FF}_{G}\left(f,a_{eff}\right)$ is the Gaussian form factor; $a_{eff}$ is the effective average scatterer size; and c is the speed of sound in breast tissue, taken here to be 1540 m/s as per measurements made using ultrasound tomography [19]. $\bar{n}$ is the average number of scatterers per unit volume and $\gamma_{0}^{2}$ is the mean square variation in acoustic impedance between the scatterers and the background medium.

After calculating feature estimates for each data block within the ROI, estimates for the five ‘primary’ QUS features were calculated by averaging across all data blocks in the segmented region. By repeating the process for each frame, feature estimates were averaged across frames to obtain a final set of feature estimates representative of the tumor. Parametric maps for each of the five primary features were created by averaging estimates from overlapping data blocks at each pixel position on the original image.

A set of texture features was derived for each of these parametric maps using the gray-level co-occurrence matrix (GLCM) method. A brief overview of the approach follows; for a detailed explanation, please refer to [20]. Using methods developed in previous work [6,8], 16 GLCMs were constructed for each of the parametric maps by combining the four possible angular relationships between neighbor-pixels (0°, 45°, 90°, 135°) with four different values of step size (1, 2, 3, and 4 pixels) using a quantization of 16 gray levels. Contrast (CON), correlation (COR), homogeneity (HOM) (also known as the inverse difference moment), and energy (ENE) (also known as uniformity or angular second moment) were derived from each matrix to obtain 16 texture feature estimates for each feature. These texture features are defined as follows.

$$f_{con}=\sum_{i,j}{\left|i-j\right|}^{2}p\left(i,j\right)$$

$$f_{cor}=\sum_{i,j}\frac{\left(i-\mu_{x}\right)\left(j-\mu_{y}\right)p\left(i,j\right)}{\sigma_{x}\sigma_{y}}$$

$$f_{ene}=\sum_{i,j}p{\left(i,j\right)}^{2}$$

$$f_{hom}=\sum_{i,j}\frac{p\left(i,j\right)}{1+\left|i-j\right|}$$

where $f_{con}$, $f_{cor}$, $\;f_{ene}$, and $\;f_{hom}$ are the contrast, correlation, energy and homogeneity respectively, $p\left(i,j\right)$ is the $\left(i,j\right)$th entry in the GLCM, and all sums are performed over the number of gray levels used for quantization. The GLCM means $\mu_{x}$ and $\mu_{y}$ and the GLCM standard deviations $\sigma_{x}$ and $\sigma_{y}$ are found by calculating the mean and standard deviation of the two marginal probability matrices:

$$p_{x}\left(i\right)=\sum_{j}p\left(i,j\right)$$

$$p_{y}\left(j\right)=\sum_{i}p\left(i,j\right)$$

These feature sets were averaged to obtain an overall estimate of each texture feature. Altogether a set of 25 features, 5 of them mean ‘primary’ QUS features and 20 of texture features, were extracted from each set of ultrasound RF data for each patient.

### 2.3. Statistical Analysis

In order to evaluate the overall level of feature estimate agreement between estimates obtained from the Sonix RP images and those obtained from the Clarius L15HD images, statistical testing was performed for each of the primary QUS features (mid-band fit, spectral slope, spectral intercept, average scatterer diameter, and average acoustic concentration) and each texture feature (contrast, correlation, homogeneity, and energy) for three sets of data: feature estimates from the pre-treatment ultrasound images (‘baseline features’), feature estimates form the week 4 time point images (‘week 4 features’), and the differences between baseline and week 4 feature estimates (‘feature differences’). Employing methods developed in previous investigations [6], the nonparametric Wilcoxon signed-rank test was used to test for statistical agreement and compute associated *p*-values for each of the three feature sets.

In addition, the Bland–Altman method [21] was employed to graphically visualize the relationship between feature estimates for certain key QUS features. Of special interest were four of the QUS texture features which previous investigations have shown to be indicators of tumor response to chemotherapy: the spectral slope contrast (SS-CON), spectral intercept energy (SI-ENE), spectral intercept homogeneity (SI-HOM), and average scatterer diameter correlation (ASD-COR) [3].

## 3. Results

**Figure 1:**
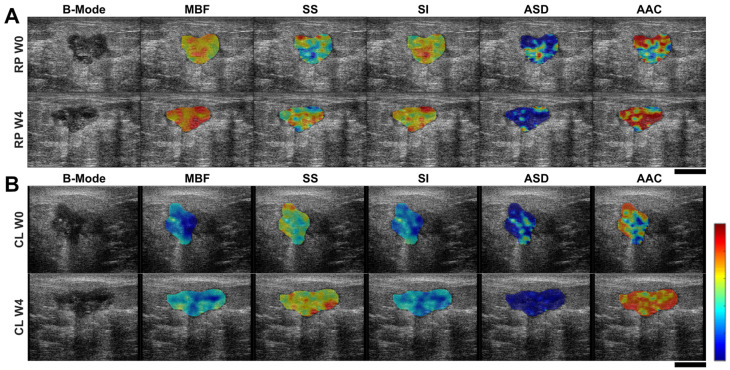
Cross-system comparison showing ultrasound B-mode images of a representative breast tumor. The tumor was imaged using both the Sonix RP (**A**) and the Clarius L15HD (**B**). Color overlays are QUS feature parametric maps (for MBF, SS, SI, ASD, and AAC) for the baseline ‘week 0’ time point (top of (**A**,**B**)) and the week 4 time point (bottom of (**A**,**B**)). The color scale shows variation in feature magnitude. The black scale bars represent 1 cm.

Patient demographic information is presented in Table 2 and includes tumor size as measured on pre-treatment imaging, pre-treatment tumor grade, and hormone receptor status for estrogen receptor (ER), progesterone receptor (PR), and human epidermal growth factor receptor 2 (HER2). For receptor status, a plus sign (+) indicates positive status and a minus sign (−) indicates negative status. The age range of patients was 37–82 years, with a median age of 53 years. The breast tumor sizes, taken to be the longest tumor dimension based on diagnostic ultrasound imaging, ranged from 1.3 cm to 7.3 cm with a median size of 3.5 cm. Two patients were classified as non-responders to chemotherapy (cases 1, 20); all others were classified as responders. Examples of QUS parametric maps for representative Clarius L15HD and Sonix RP ultrasound images are presented in Figure 1.

Cross-system statistical significance testing of each of the 25 QUS features was performed for baseline features, week 4 features, and feature differences. The *p*-values are recorded in Table 3. The range of *p*-values was 1.73 × 10^−6^ to 0.382 for the baseline features, 2.37 × 10^−5^ to 0.992 for the week 4 features, and 1.73 × 10^−6^ to 0.926 for the feature differences. For the feature differences, all five of the primary QUS features (MBF, SS, SI, ASD, AAC) were found to be in agreement at the 5% confidence level between data from the two scanners. For the baseline feature set and week 4 feature set, 0 out of 5 and 3 out of 5 of the primary features were found to be similar, respectively. Of the 20 QUS texture features examined, the number and proportion of the total for each feature set which were not found to exhibit statistically significant differences (but rather similarity) in the sample medians at the 5% confidence level were as follows. For the baseline features, 2 out of 20 (10%), for the week 4 features, 17 out of 20 (85%), and for the feature differences, 12 out of 20 (60%). The specific texture features found to be in agreement varied between feature sets.

**Figure 2:**
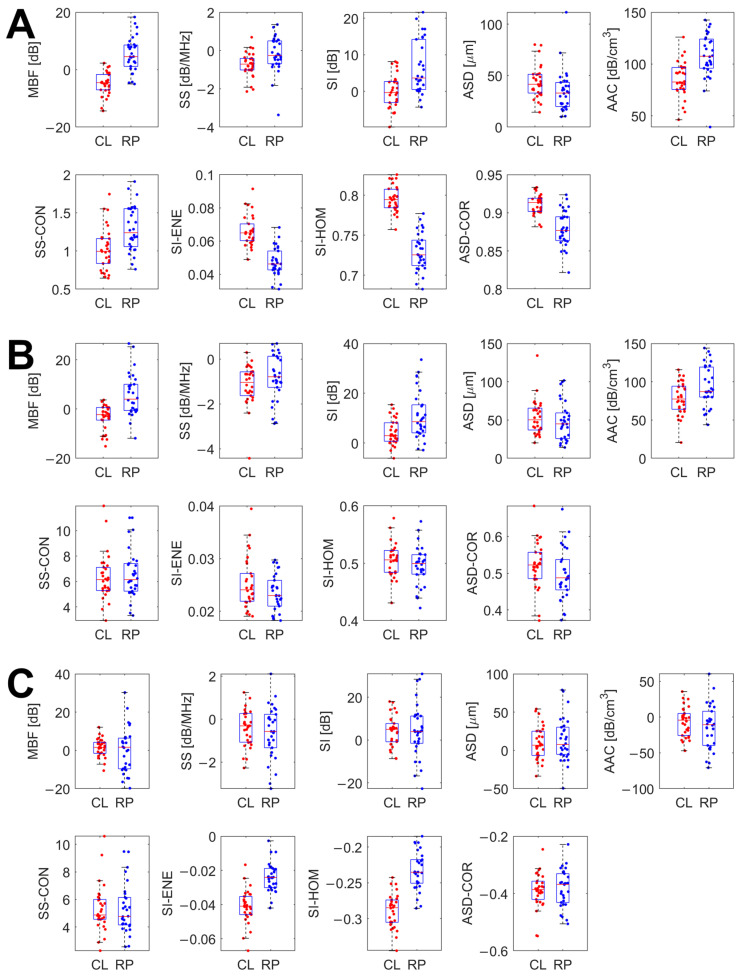
Inter-system comparison scatterplots for primary QUS features and texture feature estimates for the Clarius L15HD (in red) and Sonix RP (in blue) RF data, for the baseline features (**A**), week 4 features (**B**), and feature differences (**C**).

Scatter plots for mean QUS and key texture features of baseline features, week 4 features and feature differences for breast masses characterized using the Sonix RP and Clarius L15HD ultrasound systems are presented in Figure 2. The figure allows for comparison of feature estimate distributions between Clarius and RP-derived features for each of the three feature sets. In the box plots, the central red line indicates the median value, while the bottom and top edges represent the 25th and 75th percentiles, respectively.

**Figure 3:**
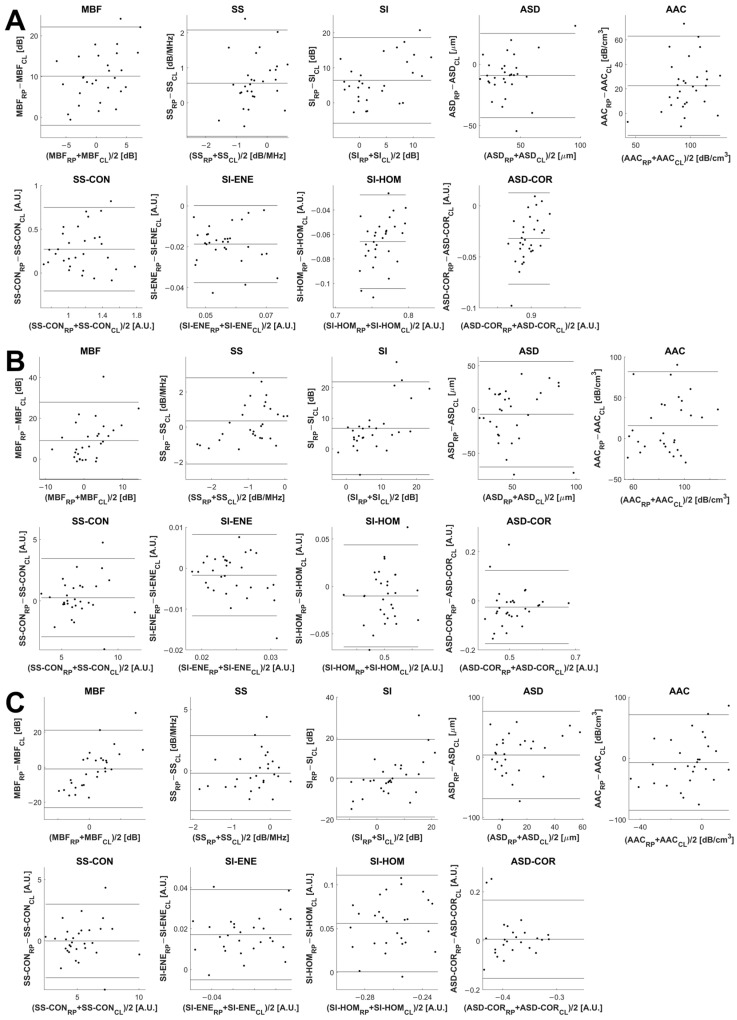
Bland–Altman plots comparing QUS and texture features acquired from breast lesions using the Sonix RP (RP) and Clarius L15 HD (CL) ultrasound systems, plotting the difference in feature estimates between systems versus the inter-system mean estimate for the baseline features (**A**), week 4 features (**B**), and feature differences (**C**). The limits of agreement for feature estimates are ±1.96 standard deviations from the mean difference.

Bland–Altman plots for some representative features are displayed in Figure 3, with horizontal lines demarcating the mean difference and limits of agreement. The limits of agreement between parameter estimates from the two clinical systems were defined by the mean difference ±1.96 standard deviations of the difference.

## 4. Discussion

Wireless portable ultrasound devices have attracted increasing interest from clinicians [22] as they have a few advantages compared to bulkier cart-bound devices. Their smaller form factor and inherent portability mean that the operator can bring the device to the patient, greatly increasing flexibility. Lower costs compared to cart-based systems may translate into increased accessibility to ultrasound imaging and associated QUS-based clinical applications. At the time of writing, the most significant disadvantage of handheld ultrasound systems is limitations on transducer sizes, sampling rates, and overall image quality imposed by miniaturization.

Previous investigations comparing QUS feature estimates produced using RF data collected using different ultrasound systems and transducers [3] have indicated that despite the use of analysis techniques and strategies aimed at improving the level of agreement between systems such as performing spectral analysis on a common bandwidth, there are still significant differences between cross-system feature estimates made for specific tumors and significant statistical differences between feature estimate distributions. These differences are attributable to variability introduced while performing the scan and to hardware and transducer property differences which are theoretically possible to eliminate using the reference phantom technique but which in practice introduce significant variation in feature estimates between systems, even when ultrasound scans are performed by the same operator at the same time point.

Prior work investigating techniques for predicting tumor response to chemotherapy using QUS features and texture features have taken the approach of calculating the differences between features extracted at different time points over the course of the patient’s neoadjuvant chemotherapy regimen and a set of pre-treatment ‘baseline’ features [3], the theory being that changes in the tissue microstructure related to cell death will correlate with changes (or lack thereof) in QUS feature values. Therefore, this study sought to examine the level of agreement not only in system feature estimates at different time points but also the level of agreement of inter-time point feature differences.

For the five primary QUS features (MBF, SS, SI, ASD, AAC), there was better agreement between ultrasound systems for the differences in feature estimates than there was for either the baseline or week 4 feature sets individually. For the QUS texture features it was the week 4 feature set that demonstrated the best level of cross-system agreement, with all but three of the texture features (MBF-CON, SI-HOM, ASD-COR) showing statistical agreement at the 5% level. The feature differences were second, with 60% of texture features in agreement, while the baseline feature set was found to have the lowest level of agreement with none of the primary QUS features and only two of the texture features (ASD-ENE and AAC-ENE) demonstrating statistical equivalence. Overall, the results indicated good agreement between system feature difference estimates for the primary features and moderate agreement for the texture features.

Of the ‘special interest’ texture features, the SS-CON and ASD-COR estimates for the feature differences did not demonstrate significant disagreement between systems at the 5% level, while the SI-ENE and SI-HOM were found to be in disagreement. Examination of the feature estimate scatterplots (Figure 2C) and the Bland–Altman plots (Figure 3C) provides graphical confirmation of significant differences in feature estimate distributions for these texture features.

The significant increase in the level of agreement in feature estimates between baseline and week four feature sets between the two scanners may be explained by the fact that for the majority of patients the size of the tumor significantly decreased after four weeks of neoadjuvant chemotherapy. Examining the ROI size metrics in Table 4 shows that the average baseline tumor ROI is roughly twice the size of the average week four tumor ROI. The exact regions of each tumor imaged with each ultrasound device and then selected for analysis were not identical; for the sake of expediency the feature estimates presented in this study are representative averages of features and texture features taken from 4 to 6 frames of ultrasound data spread out across the tumor volume. A possible result of this methodology is that the larger and more heterogeneous the average tumor is, the greater the overall variability in QUS feature estimates, and the smaller and more homogenous the average tumor is, the lower the variability. This is because larger tumors are more likely to have a heterogeneous tissue composition and hence an increased variability of feature estimates attributable to tissue heterogeneity [6].

## 5. Conclusions

This study quantified and assessed the level of agreement in QUS feature estimates produced using a cart-based and a handheld ultrasound system. Sets of feature estimates were produced for baseline and week 4 time points, as well as the differences between them, in order to investigate the changes in QUS feature estimates of LABC which occur over the course of neoadjuvant chemotherapy. A moderate level of agreement between sets of feature differences produced using the two systems was found, with all five of the primary QUS features and 60% of the 20 QUS texture features demonstrating no statistically significant differences in sample medians at the 5% confidence level. Graphical techniques were employed to confirm and visualize the level of agreement between feature sets. These findings demonstrate that the QUS feature estimation techniques which have shown great potential for use in tumor response monitoring and other clinical applications can be used with handheld ultrasound scanners such as the Clarius L15HD, but further investigation of possible techniques for improving the level of inter-system agreement of feature estimates is necessary.

Assessing the inter-system agreement of feature sets obtained at additional time points and the corresponding feature differences is a potential subject of further study and would serve to augment the observations made in the present study. Recent QUS studies [8] have also begun investigating second-order or ‘texture of texture’ features produced by constructing parametric maps of QUS texture features. The assessment of inter-system agreement for texture-of-texture feature estimates is another avenue of future investigation which will be pursued.

## Figures and Tables

**Table 1:** Summary of ultrasound transducer and imaging parameters.

Parameters	RP (L14-5W/60)	CL15
Transducer Parameters		
Number of Elements	128	192
Center Frequency [MHz]	6.3	6.9
Frequency Bandwidth range [MHz]	3–8	5.1–8.3
Imaging Parameters		
Sampling Rate [MHz]	40	30
Focal Position [mm]	17.5	20.03
Number of Scan Lines	510	192
RF Samples per Line	2064	1568

**Table 2:** Summary of patient characteristics. Horizontal lines demarcating the mean difference and limits of agreement.

ID	Age	Mass Size (cm)	Side	Grade	Histology	ER	PR	HER2
1	63	1.8	L	2	Invasive ductal carcinoma (IDC)	+	+	+
2	65	2.7	R	2	IDC	+	−	−
3	65	6.3	R	3	IDC	−	−	+
4	54	2.3	R	2	IDC	+	+	+
5	63	1.6	R	1	IDC with apocrine features	−	−	−
6	46	2.8	R	2	IDC	+	+	−
7	53	5.7	L	3	Micro-invasive carcinoma	+	−	−
8	53	6.4	R	2	IDC	−	−	+
9	53	2.6	L	2	IDC	−	−	−
10	49	1.3	R	2	Invasive breast carcinoma with papillary features	+	+	+
11	72	2.2	L	3	IDC	−	−	−
12	56	3.5	R	2	IDC	+	+	+
13	37	2.8	L	3	IDC	+	+	−
14	63	5.5	L	2	Mucinous carcinoma	+	+	−
15	37	6.1	L	3	IDC	+	+	+
16	49	6.1	R	3	IDC	+	−	+
17	46	7.3	R	2	Invasive carcinoma with extensive necrosis	−	−	−
18	50	3.0	R	2	IDC	−	−	−
19	49	5.2	R	2	IDC	+	+	−
20	53	3.3	R	3	IDC	+	−	+
21	70	6.7	L	3	Invasive lobular carcinoma (ILC)	+	+	−
22	51	7.0	L	2	IDC	−	−	−
23	70	5.4	L	2	Mixed ductal and metaplastic carcinoma	−	−	−
24	45	3.2	R	3	IDC	+	+	+
25	48	4.5	R	2	IDC	+	+	+
26	67	3.6	L	3	IDC	−	−	+
27	56	2.0	R	2	IDC	−	−	−
28	48	4.5	R	3	IDC	+	+	+
29	81	2.5	L	3	ILC	+	−	+
30	82	3.7	L	3	IDC	−	−	+

**Table 3:** Results of cross-system statistical comparison.

QUS Feature	*p*-Value, Baseline Features	*p*-Value, Week 4 Features	*p*-Value, Feature Differences
MBF	1.92 × 10^−6^ (*)	2.37 × 10^−5^ (*)	0.393
SS	6.64 × 10^−4^ (*)	0.299	0.205
SI	2.37 × 10^−5^ (*)	3.72 × 10^−5^ (*)	0.734
ASD	6.04 × 10^−3^ (*)	0.600	0.298
AAC	1.36 × 10^−5^ (*)	0.0519	0.221
MBF-con	2.88 × 10^−6^ (*)	0.0300 (*)	0.165
MBF-cor	4.07 × 10^−5^ (*)	0.245	0.530
MBF-ene	5.75 × 10^−6^ (*)	0.586	1.15 × 10^−4^ (*)
MBF-hom	1.73 × 10^−6^ (*)	0.0545	1.49 × 10^−5^ (*)
SS-con	7.69 × 10^−6^ (*)	0.465	0.877
SS-cor	3.11 × 10^−5^ (*)	0.125	0.877
SS-ene	1.92 × 10^−6^ (*)	0.136	3.88 × 10^−6^ (*)
SS-hom	1.73 × 10^−6^ (*)	0.0544	1.73 × 10^−6^ (*)
SI-con	2.13 × 10^−6^ (*)	0.393	0.688
SI-cor	6.32 × 10^−5^ (*)	0.0786	0.719
SI-ene	1.73 × 10^−6^ (*)	0.106	3.18 × 10^−6^ (*)
SI-hom	1.73 × 10^−6^ (*)	0.0449 (*)	2.13 × 10^−6^ (*)
ASD-con	1.71 × 10^−3^ (*)	0.572	0.703
ASD-cor	4.28 × 10^−6^ (*)	6.42 × 10^−3^ (*)	0.926
ASD-ene	0.0627	0.0752	0.360
ASD-hom	1.89 × 10^−4^ (*)	0.206	8.31 × 10^−4^ (*)
AAC-con	1.36 × 10^−4^ (*)	0.992	0.229
AAC-cor	1.73 × 10^−6^ (*)	0.0752	0.750
AAC-ene	0.382	0.829	0.371
AAC-hom	7.69 × 10^−6^ (*)	0.658	1.74 × 10^−4^ (*)

(*) indicates a significant difference in sample medians at the 5% confidence level.

**Table 4:** Mean and median sizes of tumor ultrasound image ROIs.

Feature Set	Mean ROI Size (Number of Windows)	Standard Deviation	Median ROI Size (Number of Windows)
CL15 baseline	913	796	678
RP baseline	1005	789	840
CL15 week 4	491	570	317
RP week 4	610	749	376

## Data Availability

The data presented in this study are available on request from the corresponding author in accordance with the institutional policies of Sunnybrook Research Institute.
